# Platelet inhibition strategies in rescue stenting after failed thrombectomy: a large retrospective multicenter registry

**DOI:** 10.1177/17562864251360913

**Published:** 2025-08-21

**Authors:** Aikaterini Anastasiou, Alex Brehm, Johannes Kaesmacher, Adnan Mujanovic, Marta de Dios Lascuevas, Tomás Carmona Fuentes, Alfonso López-Frías, Blanca Hidalgo Valverde, Ansgar Berlis, Christoph J. Maurer, Thanh N. Nguyen, Mohamad Abdalkader, Piers Klein, Guillaume Thevoz, Patrik Michel, Bruno Bartolini, Marius Kaschner, Daniel Weiss, Andrea M. Alexandre, Alessandro Pedicelli, Paolo Machi, Gianmarco Bernava, Shuntaro Kuwahara, Kazutaka Uchida, Jason Wenderoth, Anirudh Joshi, Grzegorz Karwacki, Manuel Bolognese, Agostino Tessitore, Sergio Lucio Vinci, Amedeo Cervo, Claudia Rollo, Ferdinand Hui, Aaisha Siddiqua Mozumder, Daniele Giuseppe Romano, Giulia Frauenfelder, Nitin Goyal, Vivek Batra, Violiza Inoa, Christophe Cognard, Matúš Hoferica, Riitta Rautio, Daniel P. O. Kaiser, Johannes C. Gerber, Julian Clarke, Michael R. Levitt, Marcel N. Wolf, Ahmed E. Othman, Luca Scarcia, Erwah Kalsoum, Diana Melancia, Diana Aguiar de Sousa, Maria Porzia Ganimede, Vittorio Semeraro, Flavio Giordano, Massimo Muto, Aristeidis Katsanos, Umesh Bonala, Anil M. Tuladhar, Sjoerd F. M. Jenniskens, Victoria Hellstern, Ilka Kleffner, Paolo Remida, Susanna Diamanti, Leonardo Renieri, Elena Ballabio, Luca Valvassori, Nikki Rommers, Mira Katan, Victor Schulze-Zachau, Marios-Nikos Psychogios

**Affiliations:** Diagnostic & Interventional Neuroradiology Department, University Hospital Basel, Basel, Switzerland; Diagnostic & Interventional Neuroradiology Department, University Hospital Basel, Basel, Switzerland; Department of Diagnostic and Interventional Neuroradiology, Inselspital University Hospital Bern, University of Bern, Bern, Switzerland; Diagnostic and Interventional Neuroradiology, CIC-IT 1415, CHRU de Tours, Tours, France; Department of Diagnostic and Interventional Neuroradiology, Inselspital University Hospital Bern, University of Bern, Bern, Switzerland; Interventional Neuroradiology, Vall d’Hebron University Hospital, Barcelona, Spain; Interventional Neuroradiology, Vall d’Hebron University Hospital, Barcelona, Spain; Interventional Neuroradiology, Hospital Clínico San Carlos, Madrid, Spain; Stroke Unit, Department of Neurology, Hospital Clínico San Carlos, Madrid, Spain; Diagnostic and Interventional Neuroradiology, University Hospital Augsburg, Augsburg, Germany; Diagnostic and Interventional Neuroradiology, University Hospital Augsburg, Augsburg, Germany; Department of Radiology, Boston Medical Center, Boston University Chobanian & Avedisian School of Medicine, Boston, MA, USA; Department of Radiology, Boston Medical Center, Boston University Chobanian & Avedisian School of Medicine, Boston, MA, USA; Department of Radiology, Boston Medical Center, Boston University Chobanian & Avedisian School of Medicine, Boston, MA, USA; Stroke Center, Neurology Service, Department of Clinical Neurosciences, Lausanne University Hospital, Lausanne, Switzerland; Stroke Center, Neurology Service, Department of Clinical Neurosciences, Lausanne University Hospital, Lausanne, Switzerland; Stroke Center, Neurology Service, Department of Clinical Neurosciences, Lausanne University Hospital, Lausanne, Switzerland; Department of Diagnostic and Interventional Radiology, Medical Faculty and University Hospital Düsseldorf, Heinrich-Heine-University Düsseldorf, Düsseldorf, Germany; Department of Diagnostic and Interventional Radiology, Medical Faculty and University Hospital Düsseldorf, Heinrich-Heine-University Düsseldorf, Düsseldorf, Germany; UOSA Neuroradiologia Interventistica, Fondazione Policlinico Universitario A. Gemelli IRCCS, Roma, Italy; UOSA Neuroradiologia Interventistica, Fondazione Policlinico Universitario A. Gemelli IRCCS, Roma, Italy; Università Cattolica del Sacro Cuore, Roma, Italy; Division of Neuroradiology, Geneva University Hospitals, Geneva, Switzerland; Division of Neuroradiology, Geneva University Hospitals, Geneva, Switzerland; Department of Neurosurgery, Hyogo Medical University, Nishinomiya, Japan; Department of Neurosurgery, Hyogo Medical University, Nishinomiya, Japan; Institute of Neurological Sciences, Prince of Wales Hospital, Randwick, NSW, Australia; Prince of Wales Clinical School, University of New South Wales, Sydney, NSW, Australia; Institute of Neurological Sciences, Prince of Wales Hospital, Randwick, NSW, Australia; Prince of Wales Clinical School, University of New South Wales, Sydney, NSW, Australia; Department of Radiology and Nuclear Medicine, Cantonal Hospital Lucerne, Lucerne, Switzerland; Neurocenter, Cantonal Hospital of Lucerne, Lucerne, Switzerland; Neuroradiology Unit, University Hospital A.O.U. “G. Martino”—Messina, Messina, Italy; Neuroradiology Unit, University Hospital A.O.U. “G. Martino”—Messina, Messina, Italy; Department of Biomedical, Dental and Morphological and Functional Imaging (BIOMORF), University of Messina, Messina, Italy; Department of Neuroradiology of Grande Ospedale Metropolitano Niguarda, Milan, Italy; Department of Neuroradiology of Grande Ospedale Metropolitano Niguarda, Milan, Italy; Neuroscience Institute, The Queen’s Medical Center, University of Hawaii, Honolulu, HI, USA; Neuroscience Institute, The Queen’s Medical Center, University of Hawaii, Honolulu, HI, USA; Unit of Interventional Neuroradiology, University Hospital AOU Salerno, Salerno, Italy; Unit of Interventional Neuroradiology, University Hospital AOU Salerno, Salerno, Italy; Department of Neurology, University of Tennessee Health Science Center, Memphis, TN, USA; Department of Neurological Surgery, Semmes-Murphey Clinic, Memphis, TN, USA; Department of Neurology, University of Tennessee Health Science Center, Memphis, TN, USA; Department of Neurological Surgery, Semmes-Murphey Clinic, Memphis, TN, USA; Department of Neurology, University of Tennessee Health Science Center, Memphis, TN, USA; Department of Neurological Surgery, Semmes-Murphey Clinic, Memphis, TN, USA; Neuroradiology Department, Toulouse University Hospital, Toulouse, France; Neuroradiology Department, Toulouse University Hospital, Toulouse, France; Turku University Hospital and University of Turku, Turku, Finland; Institute of Neuroradiology, Technische Universität Dresden, University Hospital Carl Gustav Carus, Dresden, Germany; Dresden Neurovascular Center, Faculty of Medicine and University Hospital Carl Gustav Carus, Technische Universität Dresden, Dresden, Germany; Institute of Neuroradiology, Technische Universität Dresden, University Hospital Carl Gustav Carus, Dresden, Germany; Dresden Neurovascular Center, Faculty of Medicine and University Hospital Carl Gustav Carus, Technische Universität Dresden, Dresden, Germany; Department of Neurological Surgery, University of Washington, Seattle, WA, USA; Department of Neurological Surgery, University of Washington, Seattle, WA, USA; Department of Neuroradiology, University Medical Center Mainz, Johannes Gutenberg University, Mainz, Germany; Department of Neuroradiology, University Medical Center Mainz, Johannes Gutenberg University, Mainz, Germany; Department of Neuroradiology, Henri Mondor Hospital, Créteil, France; Department of Neuroradiology, Henri Mondor Hospital, Créteil, France; Stroke Center, Department of Neurosciences, Lisbon Central University Hospital—ULS São José, Lisbon, Portugal; Institute of Pharmacology and Neurosciences, Faculty of Medicine, University of Lisbon, Lisbon, Portugal; Faculdade de Medicina, Universidade de Lisboa, and Gulbenkian Institute for Molecular Medicine, Lisbon, Portugal; Stroke Center, Department of Neurosciences, Lisbon Central University Hospital—ULS São José, Lisbon, Portugal; Institute of Pharmacology and Neurosciences, Faculty of Medicine, University of Lisbon, Lisbon, Portugal; Diagnostic and Interventional Neuroradiology, SS. Annunziata Hospital, Taranto, Italy; Diagnostic and Interventional Neuroradiology, SS. Annunziata Hospital, Taranto, Italy; Neuroradiology Department, AORN Cardarelli, Naples, Italy; Neuroradiology Department, AORN Cardarelli, Naples, Italy; Division of Neurology, McMaster University and Population Health Research Institute, Hamilton, ON, Canada; Division of Neurology, McMaster University and Population Health Research Institute, Hamilton, ON, Canada; Department of Neurology, Radboud University Medical Center, Nijmegen, The Netherlands; Donders Institute for Brain, Cognition and Behaviour, Radboud University, Nijmegen, The Netherlands; Department of Radiology & Nuclear Medicine, Radboud University Medical Center, Nijmegen, The Netherlands; Interventional Neuroradiology Department, Radiology and Nuclear Medicine Network, Kantonsspital St. Gallen, HOCH Health Ostschweiz, Switzerland; Stroke Medicine and Neurocritical Care Palliative Care, Neurovascular Ultrasound, Knappschaft Kliniken, University Hospital Bochum, Bochum, Germany; Neuroradiology—Fondazione IRCCS San Gerardo dei Tintori, Monza, Italy; Neurology—Fondazione IRCCS San Gerardo dei Tintori, Monza, Italy; Neurovascular Interventional Unit, Careggi University Hospital, Florence, Italy; Department of Neurology, ASST Santi Paolo e Carlo, Milan, Italy; Department of Neuroradiology, ASST Santi Paolo e Carlo, Milan, Italy; Department of Clinical Research, University Hospital Basel, University of Basel, Basel, Switzerland; Department of Neurology, Stroke Center, University Hospital of Basel, Basel, Switzerland; Diagnostic & Interventional Neuroradiology Department, University Hospital Basel, Petersgraben 4, Basel 4031, Switzerland; Diagnostic & Interventional Neuroradiology Department, University Hospital Basel, Basel, Switzerland

**Keywords:** intracranial arteriosclerosis, ischemic stroke, platelet aggregation inhibitors, stents, thrombectomy

## Abstract

**Background:**

Rescue stenting (RS) is a bailout strategy for failed thrombectomy. Optimal platelet inhibition strategy after RS remains unclear.

**Objectives:**

We aimed to describe and compare different platelet inhibition strategies during/after RS.

**Design:**

Retrospective cohort study across 34 international centers.

**Methods:**

Patients with large vessel occlusion and RS after failed thrombectomy (2019–2023) were included. Periprocedural and postprocedural platelet inhibition strategies were described and compared, focusing on glycoprotein IIb/IIIa (GPIIb/IIIa) inhibitors, single antiplatelet therapy (SAPT), and dual antiplatelet therapy (DAPT). We assessed the effects of platelet inhibition strategy and potentially covariates on the primary outcome of 90-day modified Rankin Scale (mRS) using ordinal shift analysis with proportional odds models.

**Results:**

RS was performed in 589 patients (mean age 67.9 years, 60.8% male). Numerous combinations of platelet inhibitors were administered. Periprocedural GPIIb/IIIa inhibitors were used in 61.5% of patients. Postprocedural DAPT was administered to 80.5% and SAPT to 13.3%. Functional independence (mRS 0–2) was achieved in 40.7%, while 26.3% died within 90 days. Stent occlusion occurred in 20.5%, with 67.6% of these occlusions within 24 h. Postprocedural stent-occlusion was independently associated with worse functional outcome at 90 days (OR 4.1, 95% CI 2.3–7.2, *p* < 0.001). No significant association between periprocedural GPIIb/IIIa inhibitors, and 90-day mRS or stent occlusion was found. Postprocedural SAPT was associated with worse functional outcomes (adjusted odds ratio (aOR) 2.4, 95% CI 1.1–5.0, *p* = 0.02), higher mortality (aOR 2.1, 95% CI 1.05–4.0, *p* = 0.03), and increased stent occlusion rates (aOR 4.8, 95% CI 2.3–9.7, *p* < 0.001) compared to postprocedural DAPT. Symptomatic intracranial hemorrhage occurred in 6.8% of patients, with no significant difference between antiplatelet regimens.

**Conclusion:**

Extensive heterogeneity exists in platelet inhibition strategies following RS. Stent occlusion is associated with worse clinical outcomes, and the first 24 h post-RS are critical for stent patency. Compared to SAPT, DAPT was associated with better functional outcome, lower mortality, and lower stent occlusion rates.

## Plain language summary

When clot removal for stroke fails and a stent is placed, different blood thinner treatments affect risk of stent blockage and outcomes

When doctors try to remove a blood clot during a stroke but are unsuccessful, they sometimes place a stent to reopen the blocked artery—a procedure called “rescue stenting.” After placing a stent, patients need blood-thinning medications to prevent the stent from becoming blocked again. However, the best way to manage these medications is unclear. In this study, we looked at 589 patients from 34 hospitals around the world who had rescue stenting between 2019 and 2023. We compared different strategies for using blood thinners, including strong drugs given during the procedure and either one (“single”) or two (“dual”) blood thinners given afterward. We found a lot of variation in how doctors used these medications. Using a strong blood thinner during the procedure didn’t seem to change long-term recovery or the risk of the stent blocking. However, after the procedure, patients who were treated with two blood thinners did better than those who got only one. Patients on dual therapy were more likely to recover well, less likely to die, and less likely to have their stent block again. Importantly, most stent blockages happened within the first 24 hours after the procedure, and patients with stent blockages did worse, highlighting the 24h period as critical for blood thinner treatment.

## Introduction

Stroke significantly contributes to morbidity and mortality worldwide.^1^ Mechanical recanalization of the occluded artery by thrombectomy has been shown to benefit patients, reducing disability and mortality.^2^ Successful reperfusion is a strong predictor regarding functional outcome and survival.^3,4^ However, in 10%–30% of patients, thrombectomy fails to achieve successful reperfusion^5,6^ with the majority of cases attributed to intracranial atherosclerotic disease (ICAD).^7,8^ Failed reperfusion has been associated with an increased risk of early neurologic deterioration,^9^ and poor reperfusion was shown to be associated with worse outcomes compared to best medical management.^10^

Rescue stenting (RS) has been proposed as a bail-out strategy in failed thrombectomy cases and nonrandomized data suggest that RS might be a safe and effective option after failed thrombectomy.^11–17^ While one trial in China did not show better outcomes after bailout angioplasty or stenting in patients with unsuccessful recanalization or at risk of reocclusion after thrombectomy,^18^ the ongoing IntraCranial Atherosclerosis Related Large-vessel Occlusion Treated With Urgent Stenting trial (ICARUS, NCT06472336) is currently assessing whether early RS is superior to continued mechanical thrombectomy in ICAD-related failed reperfusion. However, antiplatelet medication is needed when RS is performed. Numerous management options regarding the platelet inhibition strategy exist^19^: Glycoprotein IIb/IIIa (GPIIb/IIIa) inhibitors may be used, single or dual oral antiplatelets may be administered, and the dosage may be adapted. Interventionalists must balance the risk of stent occlusion against the possibility of hemorrhagic complications. Currently, no high-quality data exist to draw conclusions regarding the optimal antiplatelet strategy.

This study aims to describe the antiplatelet strategies utilized in multiple international centers and compare different platelet inhibition strategies in RS after failed thrombectomy in large vessel occlusions (LVOs) using data from a large, international, multicenter registry.

## Methods

In this retrospective cohort study, we performed an analysis of the “Blood pressure and Antiplatelet medication management after rescue angioplasty after failed Endovascular treatment in Large and distal vessel occlusions with probable IntraCranial Atherosclerotic Disease” (BASEL ICAD) registry. In this international registry, 34 centers retrospectively collected patient data using the following inclusion criteria: adult patients (age 18 years and older) with acute ischemic stroke who underwent thrombectomy between January 1st, 2019, and December 31st, 2023 and in whom either RS or rescue angioplasty was performed after failed thrombectomy. For this analysis of the BASEL ICAD registry, only patients with LVO who received RS were included. No other exclusion criteria were applied. Data from participating centers were curated by reviewing patient charts and procedure notes.

The primary clinical outcome was the modified Rankin Scale (mRS) at 90 days, and mRS 0–2 was considered a good functional outcome. The primary technical outcome was the rate of intra- and postprocedural stent occlusion. Further outcomes of interest included the National Institute of Health Stroke Scale (NIHSS) at 24 h, clinical worsening (defined as increase in NIHSS ⩾ 4 points), symptomatic intracranial hemorrhage (sICH), any subarachnoid hemorrhage (SAH), all-cause mortality within 90 days and procedural complications.

Periprocedural medication was defined as initiated during the procedure. Postprocedural medication was defined as initiated after the procedure had ended. The switch from periprocedural to postprocedural medication was left at the discretion of the treating physicians, but usually was within 24 h after the procedure.

### Statistical analysis

Baseline characteristics were described as mean with standard deviation or median with interquartile range for continuous variables, and frequency with percentage for categorical variables. Regarding periprocedural medication, patients who received GPIIb/IIIa-inhibitors were compared with patients who did not receive GPIIb/IIIa-inhibitors. Regarding postprocedural medication, patients who managed with single antiplatelet therapy (SAPT) were compared to patients with dual antiplatelet therapy (DAPT). We assessed effects of the platelet inhibition strategy and potential covariates on the primary outcomes using an adjusted ordinal shift analysis with proportional odds models. Predefined covariates were prestroke mRS (3–5 vs 0–2), age, sex, intravenous thrombolysis (yes vs no), and NIHSS at admission. We adjusted for clustering of data by center by adding center as a random intercept to all models. The results are presented as the adjusted common odds ratio (cOR) (ordinal shift analysis) or adjusted OR (logistic regression) with corresponding 95% confidence interval and the uncorrected *p*-values.

As a secondary analysis, to control for potential bias by indication, we matched patients under postinterventional SAPT to patients under DAPT to control for potential bias by indication. A 1:1 nearest neighbor matching without replacement based on the Mahalanobis distance was applied using the following matching variables: age, NIHSS at admission, prestroke mRS, intravenous thrombolysis, periprocedural SAH or vessel perforation, intraprocedural stent occlusion with exact matching for the latter four variables. We compared the rate of posttreatment stent occlusion between matched SAPT and DAPT patients using a Fisher exact test.

As a supplementary analysis, we investigate the effect of stent patency status on mRS at 90 days in a mixed-effects ordinal regression model, adjusted for prestroke mRS (3–5 vs 0–2), age, sex, intravenous thrombolysis (yes vs no), and NIHSS at admission.

Statistical analysis was performed by a professional statistical analyst (N.R.) using R v4.3.2 (https://www.r-project.org/). No adjustment for multiple testing was done and *p*-values < 0.05 were deemed statistically significant. This article follows the STROBE reporting guidelines (http://www.strobestatement.org).

## Results

### Patient cohort and treatment characteristics

Overall, 589 patients met the inclusion criteria (60.8% male, mean age 67.9 ± 12.7 years). The median (IQR) baseline NIHSS was 12 (11). Intravenous thrombolysis was administered prior to EVT in 26.4% of patients. The median (IQR) time from symptom onset to groin puncture was 310 (403) min. Median (IQR) ASPECT (Alberta Stroke Program Early CT Score) Score was 9 (2). Table 1 summarizes the patient baseline characteristics. After a median of two thrombectomy passes (IQR 1–3), RS was performed in all patients. In 50.9%, balloon angioplasty was performed before stenting, while 18.0% of patients were subjected to balloon angioplasty after stenting and 8.1% of patients received balloon angioplasty both before and after stenting. Median time from onset to recanalization was 400 min (IQR 287–693 min). Supplemental Table S1 contains procedural details.

**Table 1.** Patient baseline characteristics.

Baseline characteristic	All patients (*n* = 589)
Age (years, mean ± SD)	67.9 ± 12.7
Male sex	60.8% (*n* = 358)
Cerebrovascular risk factors	
Hypertension	76.1% (*n* = 434)
Hyperlipidemia	38.0% (*n* = 208)
Dyslipidemia	34.0% (*n* = 163)
Diabetes mellitus	32.4% (*n* = 183)
Current or past smoking	36.7% (*n* = 196)
Atrial fibrillation	17.1% (*n* = 96)
History of stroke or transitory ischemic attack	24.9% (*n* = 136)
Prestroke mRS	
0	65.8% (*n* = 383)
1	22.5% (*n* = 131)
2	6.9% (*n* = 40)
3	4.0% (*n* = 23)
4	0.7% (*n* = 4)
5	0.2% (*n* = 1)
Prestroke anticoagulation use	14.9% (*n* = 77)
Prestroke antiplatelet use	
None	67.3% (*n* = 346)
Single antiplatelets	25.9% (*n* = 133)
Dual antiplatelets	6.8% (*n* = 35)
NIHSS at admission (median (IQR))	12 (IQR 11, range 7-18)
Intravenous thrombolysis	26.4% (*n* = 155)
Time from onset to groin puncture in minutes (median (IQR))	310 (403)
Site of arterial occlusion	
Intracranial internal carotid artery	13.2% (*n* = 78)
M1-segment of the middle cerebral artery	55.3% (*n* = 326)
Basilar artery	23.1% (*n* = 136)
V4-segment of the vertebral artery	8.3% (*n* = 49)

mRS, modified Rankin Scale; NIHSS, National Institutes of Health Stroke Scale; IQR, Interquartile Range.

### Platelet inhibition strategies

**Figure 1.**
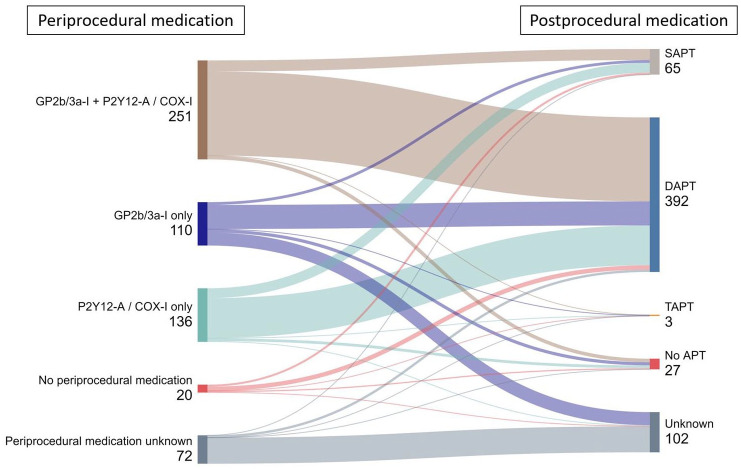
Sankey diagram shows number of patients under different antiplatelet strategies. APT, Antiplatelet therapy; COX-I, Cyclooxygenase-inhibitors, such as Aspirin; DAPT, Dual antiplatelet therapy; GP2b/3a-I, Glycoprotein-IIb/IIIa-inhibitors, such as Tirofiban, Eptifibatide, and Abciximab; P2Y12-A, P2Y12-Antagonists, such as Clopidogrel or Cangrelor; SAPT, Single antiplatelet therapy; TAPT, Triple antiplatelet therapy.

Various combinations of peri- and postprocedural medications were used. During the procedure, the majority of patients received a combination of GPIIb/IIIa-inhibitors and P2Y12-Antagonists and/or Cyclooxygenase-inhibitors (48.5%) or GPIIb/IIIa-inhibitors alone (21.3%) or P2Y12-Antagonists and/or Cyclooxygenase-inhibitors alone (26.3%). Only 3.9% of patients did not receive any periprocedural medication. After the procedure, 80.5% of patients received dual antiplatelet therapy (DAPT) and 13.3% of patients received single antiplatelet therapy (SAPT). A minority received triple antiplatelet therapy (0.6%) and 5.5% of patients did not receive any postprocedural antiplatelets. DAPT consisted of Aspirin + Clopidogrel in 62.8%, Aspirin + Ticagrelor in 34.4%, Aspirin + Prasugrel in 2.6%, and Clopidogrel + Ticagrelor in 0.2%. Figure 1 illustrates the number of patients under each platelet inhibition strategy. The Supplemental Tables S2 and S3 contain details of peri- and postinterventional antiplatelet medication.

### Outcomes

Overall, 40.7% of patients reached functional independence at 90 days, that is, mRS 0–2. All-cause mortality at 90 days was 26.3%. Overall, stent occlusion occurred in 102 (20.5%) patients. Intraprocedural stent occlusion was reported in 8.5% of patients and postprocedural stent occlusion occurred in 12.4% (45.5% within 24 h, 30.3% between 24 h and 7 days, 7.6% after 7 days and 16.7% at an unknown timepoint). Overall, 67.6% of stent occlusions occurred within 24 h after stent implantation. sICH occurred in 6.8% of patients and 19.9% experienced any SAH at any time point. In 6.1% of patients, subarachnoid hemorrhage or vessel perforation was observed during the intervention. Extracranial vessel dissection occurred in 2.0%, femoral or retroperitoneal hematoma in 2.0%, and other complications in 5.4%. The median NIHSS at 24 h was 10 (IQR 3–19).

### Comparisons regarding periprocedural medication

Overall, 361 patients received GPIIb/IIIa-inhibitors, while 156 patients were managed without GPIIb/IIIa-inhibitors. In multiple odds modeling, no independent association between the use of GPIIb/IIIa-inhibitors and a lower mRS score at 90 days was found (common OR 1.3 (95% CI 0.8, 2.0), *p* = 0.3). Periprocedural GPIIb/IIIa-inhibitors were not independently associated with rates of periprocedural stent occlusion (OR 0.8 (0.4, 1.6), *p* = 0.5), sICH (OR 1.0 (0.5–2.1], *p* = 1.0), any SAH (OR 1.1 (0.7–1.8), *p* = 0.6), or death within 90 days (OR 1.2 (0.8–2.0), *p* = 0.4).

### Comparisons regarding postprocedural medication

**Figure 2.**
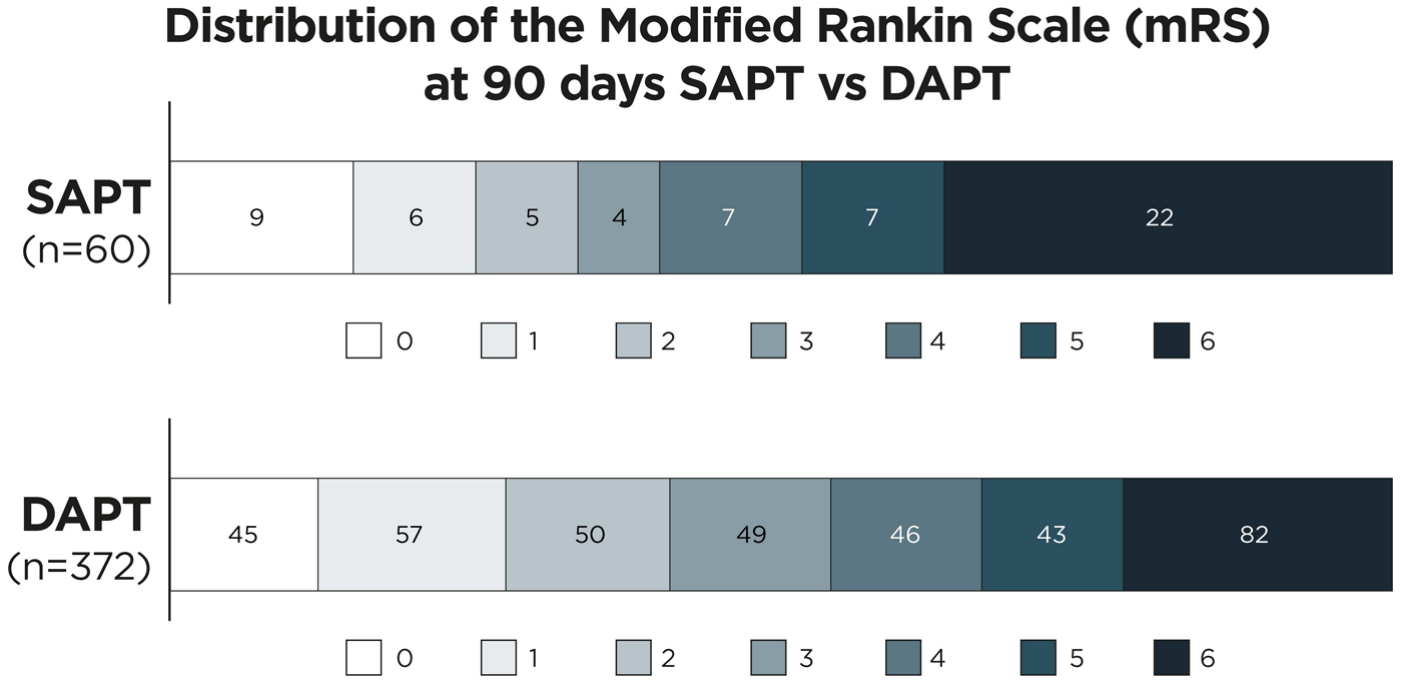
mRS of patients after rescue stenting with single versus dual postprocedural antiplatelet therapy. mRS at 90 days was available for 92.3% of SAPT patients and for 94.9% of DAPT patients. DAPT, dual antiplatelet therapy; mRS, Modified Rankin Scales; SAPT, Single antiplatelet therapy.

A total of 65 patients received postprocedural SAPT while 396 patients received DAPT. Figure 2 shows mRS bar graphs for patients under postprocedural SAPT and DAPT. Postprocedural SAPT was independently associated with worse functional outcomes (cOR 0.2 (0.1, 0.4), *p* = 0.02), a higher mortality (aOR 2.1 (1.0, 4.0), *p* = 0.03) and higher rates of postprocedural stent occlusion (OR 4.8 (2.3, 9.7), *p* < 0.001). After matching of 49 patients with postprocedural SAPT to 49 patients with postprocedural DAPT, SAPT patients had a higher rate of postinterventional stent occlusion (29.2% vs 8.7%, *p* = 0.024). The absolute rate of sICH was higher in patients under SAPT, but the adjusted model did not show a significant treatment effect (OR 2.2 (0.7, 5.5), *p* = 0.12). The same was true for SAH (OR 1.8 (0.9, 3.4), *p* = 0.07).

### Comparisons regarding stent patency status

Postprocedural stent-occlusion was independently associated with worse functional outcome at 90 days (OR 4.1 (2.3, 7.2), *p* < 0.001). Patients with postprocedural stent occlusion demonstrated clinical worsening defined as increase in NIHSS ⩾ 4 points in 74.2% of the cases. Intraprocedural stent-occlusion however was not significantly associated with higher mRS at 90 days (OR 1.7 (0.9, 3.1), *p* = 0.11).

## Discussion

In this retrospective analysis of a large multicenter registry, we report the outcomes of 589 patients who received RS as bailout therapy after failed thrombectomy. In our study, 40.7% of patients reached functional independence at 90 days, and overall mortality at 90 days was 26.3%. These results align with other retrospective studies of RS.^16^

To the best of our knowledge, this study is the first to report details of the extensive heterogeneity of different platelet inhibition strategies in rescue stenting after failed thrombectomy. Not only combinations of antiplatelet agents differed widely: heterogeneity regarding the modus of initiation (e.g., loading) and the dosage was substantial, too. While individualized patient care is desirable in general, the paucity of data on platelet inhibition after rescue stenting may suggest that this heterogeneity rather reflects uncertainty about the optimal strategy. This has important implications for the design and interpretation of recently published and ongoing trials: since rescue stenting without sufficient platelet inhibition has a high likelihood of stent reocclusion and thus failure, platelet inhibition regimens should be regarded as an integral component of the intervention. Furthermore, the lack of a consensus on optimal platelet inhibition may contain a risk of treatment disparity: in patients with lower socioeconomic status, limited financial resources could lead physicians to choose the cheapest available medication rather than what they believe to be the optimal therapy, as long as there is no evidence to guide standards of platelet inhibition post-RS. A similar pattern is reflected in a survey on the choice of platelet inhibition for rescue stenting of basilar artery occlusions, in which respondents from high-income countries more often chose GPIIb/IIIa inhibitors than respondents from middle-income countries.^20^

No significant independent associations between functional outcome at 90 days and the usage of periprocedural GPIIb/IIIa inhibitors were found. However, GPIIb/IIIa inhibitors are known as potent rescue medications in acute thrombotic scenarios.^21,22^ A possible explanation may include that intraprocedural stent occlusions occurring in patients without GPIIb/IIIa inhibitors were noticed by the interventionalist, who was able to react immediately, while postprocedural stent occlusions might depend more on postprocedural antiplatelets. Finally, this study did not demonstrate increased risks when GPIIb/IIIa inhibitors are used.

Postprocedural SAPT was less frequently administered than DAPT and was independently associated with worse functional outcome, worse survival, and higher rates of stent occlusion. However, this needs to be interpreted with caution since there is likely bias by indication. Patients with large infarcts at presentation or patients with hemorrhagic complications during the procedure might have been excluded from DAPT. Not only hemorrhagic transformation with large hematomas^23^ but also postinterventional subarachnoid hemorrhage of higher grades has been associated with poorer outcomes.^24^ Therefore, the patient status before the initiation of postprocedural antiplatelets may have contributed to worse functional outcomes. In our study, there was no independent significant association between hemorrhagic transformation or subarachnoid hemorrhage and the choice to use SAPT, but our dataset may have been underpowered to detect an existing connection. Furthermore, matching between SAPT and DAPT patients was performed to reduce the risk of bias and confirmed the association of SAPT with higher rates of postinterventional stent occlusions.

In their multicenter prospective registry, Baek et al.^13^ found an independent association between stent patency and better functional outcomes. In our data, 74% of patients with postprocedural stent occlusion developed clinical worsening and postprocedural stent occlusion was significantly associated with worse mRS at 90 days, which correspond well to the study from Baek et al. In summary, it is likely that the higher rate of stent occlusion in patients under SAPT contributed to worse functional outcomes.

In our series, 20.5% of patients experienced stent occlusions, which were associated with neurologic deterioration and worse functional outcome at 90 days. In emergent extracranial carotid stenting, stent occlusion rates of 8%–22% have been reported, and stent occlusion has also been associated with poor clinical outcome.^25,26^ Approximately two-thirds of stent occlusions occurred during the procedure or within 24 h after the procedure. This has important consequences for clinical patient management, indicating that the first 24 h of antithrombotic medication are critical for patients after RS. While postprocedure stent occlusions were associated with worse functional outcomes, intraprocedural stent occlusions were not. This may be explained by detection latency: in intraprocedural stent occlusion, the interventionalist in charge may react immediately to restore and then confirm lasting stent patency. Postprocedure stent occlusion may be unnoticed for hours, especially in patients still recovering from anesthesia and systemic application of medications is the only possible immediate response. Furthermore, real-time imaging is typically not available to verify treatment success. For these reasons, it is reasonable to assume a longer duration of hypoperfusion in postprocedure stent occlusion.

The question of whether emergent antiplatelet medication increases the risk of hemorrhagic transformation is still under debate. While the “Safety and efficacy of aspirin, unfractionated heparin, both, or neither during endovascular stroke treatment” trial (MR CLEAN-MED) found that for thrombectomy patients in general, 300 mg of periprocedural aspirin increased the risk of sICH,^27^ a meta-analysis focusing on patients with failed thrombectomy showed rescue stenting with antiplatelet therapy was not associated with a higher risk of sICH.^11^ In our study, patients receiving DAPT did not have a higher frequency of sICH compared to patients receiving SAPT. On the contrary, patients receiving SAPT had an absolute higher percentage of sICH, although this was not significant. The higher frequency of postinterventional stent occlusion in SAPT patients might have led to higher absolute infarct volumes which go along with an increased risk of sICH, although this hypothesis needs to be verified in future studies.

This study has limitations, including its nonrandomized retrospective design. The cohort may be underpowered to detect existing associations, for example, between hemorrhagic events and the choice to use GP-IIb-IIIa-inhibitors, DAPT or SAPT. Furthermore, the combination of various peri- and postinterventional medications leads to heterogeneity in the study population. On the one hand, this reflects real-world practice and therefore strengthens the study’s validity. On the other hand, for meaningful comparisons patients had to be assigned to broader groups such as postprocedural SAPT and DAPT and antiplatelet regimes were nonuniform within these groups. Finally, other strategies such as the transient deployment of a stentretriever across the lesion during infusion of GP IIb/IIIa inhibitors without permanent stenting^28^ might be therapeutic options, too, and have not been evaluated in this study.

Strengths of this study include its international multicenter design and its dimension of the patient cohort.

## Conclusion

In conclusion, platelet inhibition strategies for rescue stenting after failed thrombectomy are characterized by extensive heterogeneity. The first 24 h post-RS are critical for stent patency. In this large retrospective cohort study, DAPT was associated with better functional outcome, lower mortality, and lower stent occlusion rates compared to SAPT.

## Supplemental Material

sj-docx-1-tan-10.1177_17562864251360913 – Supplemental material for Platelet inhibition strategies in rescue stenting after failed thrombectomy: a large retrospective multicenter registry Supplemental material, sj-docx-1-tan-10.1177_17562864251360913 for Platelet inhibition strategies in rescue stenting after failed thrombectomy: a large retrospective multicenter registry by Aikaterini Anastasiou, Alex Brehm, Johannes Kaesmacher, Adnan Mujanovic, Marta de Dios Lascuevas, Tomás Carmona Fuentes, Alfonso López-Frías, Blanca Hidalgo Valverde, Ansgar Berlis, Christoph J. Maurer, Thanh N. Nguyen, Mohamad Abdalkader, Piers Klein, Guillaume Thevoz, Patrik Michel, Bruno Bartolini, Marius Kaschner, Daniel Weiss, Andrea M. Alexandre, Alessandro Pedicelli, Paolo Machi, Gianmarco Bernava, Shuntaro Kuwahara, Kazutaka Uchida, Jason Wenderoth, Anirudh Joshi, Grzegorz Karwacki, Manuel Bolognese, Agostino Tessitore, Sergio Lucio Vinci, Amedeo Cervo, Claudia Rollo, Ferdinand Hui, Aaisha Siddiqua Mozumder, Daniele Giuseppe Romano, Giulia Frauenfelder, Nitin Goyal, Vivek Batra, Violiza Inoa, Christophe Cognard, Matúš Hoferica, Riitta Rautio, Daniel P. O. Kaiser, Johannes C. Gerber, Julian Clarke, Michael R. Levitt, Marcel N. Wolf, Ahmed E. Othman, Luca Scarcia, Erwah Kalsoum, Diana Melancia, Diana Aguiar de Sousa, Maria Porzia Ganimede, Vittorio Semeraro, Flavio Giordano, Massimo Muto, Aristeidis Katsanos, Umesh Bonala, Anil M. Tuladhar, Sjoerd F. M. Jenniskens, Victoria Hellstern, Ilka Kleffner, Paolo Remida, Susanna Diamanti, Leonardo Renieri, Elena Ballabio, Luca Valvassori, Nikki Rommers, Mira Katan, Victor Schulze-Zachau and Marios-Nikos Psychogios in Therapeutic Advances in Neurological Disorders

sj-docx-2-tan-10.1177_17562864251360913 – Supplemental material for Platelet inhibition strategies in rescue stenting after failed thrombectomy: a large retrospective multicenter registry Supplemental material, sj-docx-2-tan-10.1177_17562864251360913 for Platelet inhibition strategies in rescue stenting after failed thrombectomy: a large retrospective multicenter registry by Aikaterini Anastasiou, Alex Brehm, Johannes Kaesmacher, Adnan Mujanovic, Marta de Dios Lascuevas, Tomás Carmona Fuentes, Alfonso López-Frías, Blanca Hidalgo Valverde, Ansgar Berlis, Christoph J. Maurer, Thanh N. Nguyen, Mohamad Abdalkader, Piers Klein, Guillaume Thevoz, Patrik Michel, Bruno Bartolini, Marius Kaschner, Daniel Weiss, Andrea M. Alexandre, Alessandro Pedicelli, Paolo Machi, Gianmarco Bernava, Shuntaro Kuwahara, Kazutaka Uchida, Jason Wenderoth, Anirudh Joshi, Grzegorz Karwacki, Manuel Bolognese, Agostino Tessitore, Sergio Lucio Vinci, Amedeo Cervo, Claudia Rollo, Ferdinand Hui, Aaisha Siddiqua Mozumder, Daniele Giuseppe Romano, Giulia Frauenfelder, Nitin Goyal, Vivek Batra, Violiza Inoa, Christophe Cognard, Matúš Hoferica, Riitta Rautio, Daniel P. O. Kaiser, Johannes C. Gerber, Julian Clarke, Michael R. Levitt, Marcel N. Wolf, Ahmed E. Othman, Luca Scarcia, Erwah Kalsoum, Diana Melancia, Diana Aguiar de Sousa, Maria Porzia Ganimede, Vittorio Semeraro, Flavio Giordano, Massimo Muto, Aristeidis Katsanos, Umesh Bonala, Anil M. Tuladhar, Sjoerd F. M. Jenniskens, Victoria Hellstern, Ilka Kleffner, Paolo Remida, Susanna Diamanti, Leonardo Renieri, Elena Ballabio, Luca Valvassori, Nikki Rommers, Mira Katan, Victor Schulze-Zachau and Marios-Nikos Psychogios in Therapeutic Advances in Neurological Disorders

## Appendix

### Abbreviations

BASEL ICAD registry Blood pressure and antiplatelet medication management after reScue angioplasty after failed endovascular treatment in large and distal vessel occlusions with probable IntraCranial Atherosclerotic Disease (BASEL ICAD) registry

ICAD Intracranial Atherosclerotic Disease

ICARUS trial IntraCranial Atherosclerosis-Related Large-vessel Occlusion-Treated With Urgent Stenting trial

LVO Large Vessel Occlusion

mRS Modified Rankin Scale

NIHSS National Institutes of Health Stroke Scale

RS Rescue Stenting

SAH Subarachnoid Hemorrhage

sICH Symptomatic Intracranial Hemorrhage

## Declarations

**Ethics approval and consent to participate:** This registry was approved by the applicable ethics committee (Ethics Committee Northwestern and Central Switzerland, BASEC ID 2024-00904). The need for individual patient consent was waived.

**Consent for publication:** Not applicable.

**Competing interests:** V. S.-Z. discloses speaker fees from Medtronic Inc. (money paid to institution). M.-N.P. discloses unrestricted grants from Swiss National Science Foundation (SNF), Bangerter-Rhyner Stiftung, Stryker Neurovascular Inc., Phenox GmbH, Medtronic Inc., Rapid Medical Inc., and Penumbra Inc for the DISTAL trial, grant for SPINNERS trial from Siemens Healthineers AG (money paid to institution) and the following speaker fees: Stryker Neurovascular Inc., Medtronic Inc., Penumbra Inc., Acandis GmbH, Phenox GmbH, Rapid Medical Inc. and Siemens Healthineers AG (money paid to institution). T.-N.N.: discloses Associate Editor of Stroke; Advisory board of Brainomix, Aruna Bio; speaker for Genentech, Kaneka; consulting for Medtronic. D.-G.R: discloses proctor and consultant for Penumbra, Balt Italy, Balt Europe, Terumo Europe, Striker, Wallaby Phenox. J.-C.G.: discloses local PI of RECHRUT study. AHK: discloses research support from the Heart and Stroke Foundation Canada and Canadian Institutes of Health Research, consulting fees from Diamedica Therapeutics Inc, Bayer Inc and AbbVie Inc.

**Availability of data and materials:** Anonymized data are available upon reasonable request from the corresponding author.
