# Supplementary material for: Platelet inhibition strategies in rescue stenting after failed thrombectomy: a large retrospective multicenter registry
Source: Ther Adv Neurol Disord. 2025 Aug 21;18:17562864251360913. doi: 10.1177/17562864251360913 (PMC12374045; doi:10.1177/17562864251360913)
Supplement: sj-docx-1-tan-10.1177_17562864251360913 – Supplemental material for Platelet inhibition strategies in rescue stenting after failed thrombectomy: a large retrospective multicenter registry [file sj-docx-1-tan-10.1177_17562864251360913.docx]

**Online Supplement**

| **Characteristic** | **All patients (n=589)** |
| --- | --- |
| General anesthesia | 67.3% (n=339) |
| Radial access | 10.1% (n=51) |
| Highest mTICI achieved prior to rescue therapy* |  |
| 0 | 31.5% (n=153) |
| 1 | 14.6% (n=71) |
| 2a | 14.6% (n=71) |
| 2b | 20.0% (n=97) |
| 2c | 7.2% (n=35) |
| 3 | 12.1% (n=59) |
| Final mTICI |  |
| 0 | 23.5% (n=11) |
| 1 | 15.0% (n=73) |
| 2a | 14.2% (n=69) |
| 2b | 23.7% (n=115) |
| 2c | 10.3% (n=50) |
| 3 | 13.4% (n=65) |
| Balloon angioplasty |  |
| None | 22.9% (n=135) |
| Before Stenting | 50.9% (n=300) |
| After Stenting | 18.0% (n=106) |
| Both before and after Stenting | 8.1% (n=48) |
| Balloon angioplasty only | 0% (n=0) |

**Supplement Table S1:** Intervention characteristics.

*Please note that re-occlusion after successful reperfusion may have lead to rescue stenting.

| **Periprocedural medication** | **Number of patients*** |
| --- | --- |
| Periprocedural GP-IIb/IIIa-Inhibitors |  |
| Tirofiban | 240 (46.4%) |
| Bolus of Tirofiban | 209 (40.4%) |
| Continuous infusion of Tirofiban** | 185 (35.8%) |
| Eptifibatide | 119 (23.0%) |
| Bolus of Eptifibatide | 101 (19.5%) |
| Continuous infusion of Eptifibatide** | 41 (7.9%) |
| Abciximab | 1 (0.2%) |
| Periprocedural P2Y12-Antagonists |  |
| Cangrelor | 52 (10.1%) |
| Bolus of Cangrelor | 52 (10.1%) |
| Continuous infusion of Cangrelor** | 51 (9.9%) |
| Clopidogrel | 164 (31.7%) |
| < 300 mg | 14 (2.7%) |
| 300 mg | 118 (22.8%) |
| > 300 mg | 32 (6.2%) |
| Ticagrelor | 49 (9.5%) |
| < 180 mg | 7 (1.4%) |
| 180 mg | 39 (7.5%) |
| > 180 mg | 2 (0.4%) |
| Ticagrelor dosage unknown | 1 (0.2%) |
| Prasugrel | 8 (1.5%) |
| < 60 mg | 8 (1.5%) |
| Periprocedural COX-I-Inhibitors |  |
| Aspirin | 236 (45.6%) |
| < 300 mg | 39 (7.5%) |
| 300 mg | 28 (5.4%) |
| > 300 mg | 169 (32.7%) |
| No periprocedural antiplatelet medication | 20 (3.9%) |

**Table S2:** Periprocedural antiplatelet medication

GP-IIb/IIIa-Inhibitors: Glycoprotein-IIb/IIIa-Inhibitors. COX-I: Cyclooxygenase-Inhibitors. *Periprocedural medication was unknown for 72 patients. **Continuous infusions of intravenous antiplatelets were administered either after application of an intravenous bolus or without bolus.

| **Postprocedural medication** | **Number of patients*** |
| --- | --- |
| Postprocedural P2Y12-Antagonists |  |
| Clopidogrel | 289 (59.3%) |
| 75 mg/d | 287 (58.9%) |
| > 75 mg/d | 1 (0.2%) |
| Clopidogrel dosage unknown | 1 (0.2%) |
| Ticagrelor | 142 (29.2%) |
| 90 mg/d | 3 (0.6%) |
| 180 mg/d | 139 (28.5%) |
| Prasugrel | 10 (2.1%) |
| 5 mg/d | 4 (0.8%) |
| 10 mg/d | 5 (1.0%) |
| > 10 mg/d | 1 (0.2%) |
| Postprocedural COX-I-Inhibitors |  |
| Aspirin | 418 (85.8%) |
| < 100 mg/d | 53 (10.9%) |
| 100 mg/d | 340 (69.8%) |
| > 100 mg/d | 24 (4.9%) |
| Aspirin dosage unknown | 1 (0.2%) |
| SAPT | 65 (13.3%) |
| DAPT | 392 (80.5%) |
| TAPT | 3 (0.6%) |
| No postprocedural medication | 27 (5.5%) |

**Table S3:** Postprocedural antiplatelet medication

*Postprocedural medication was unknown for 102 patients. COX-I: Cyclooxygenase-Inhibitors. SAPT: Single antiplatelet therapy. DAPT: Dual antiplatelet therapy. TAPT: Triple antiplatelet therapy.
